# Modulator Therapy in Cystic Fibrosis Patients with *cis* Variants in F508del Complex Allele: A Short-Term Observational Case Series

**DOI:** 10.3390/jpm12091421

**Published:** 2022-08-31

**Authors:** Vito Terlizzi, Claudia Centrone, Beatrice Ferrari, Chiara Castellani, Tarini N. A. Gunawardena, Giovanni Taccetti, Onofrio Laselva

**Affiliations:** 1 Department of Paediatric Medicine, Cystic Fibrosis Regional Reference Center, Meyer Children’s Hospital, 50139 Florence, Italy; 2 Diagnostic Genetics Unit, Careggi University Hospital, 50134 Florence, Italy; 3 Rehabilitation Unit, Meyer Children’s Hospital, 50139 Florence, Italy; 4 Department of Radiology, Meyer Children’s Hospital, 50139 Florence, Italy; 5 Programme in Molecular Medicine, The Hospital for Sick Children, Toronto, ON M5G 8X4, Canada; 6 Programme in Translational Medicine, The Hospital for Sick Children, Toronto, ON M5G 8X4, Canada; 7 Department of Clinical and Experimental Medicine, University of Foggia, 71122 Foggia, Italy

**Keywords:** cystic fibrosis, CFTR, complex allele, modulators, correctors

## Abstract

Previous studies reported the influence of *cis* variants in F508del cystic fibrosis (CF) patients in their responses to *CFTR* modulators. The current study is a prospective, observational study involving three patients with CF and pancreatic insufficiency, carrying a complex allele including F508del with A238V, I1027T, or L467F. We report clinical data before and after 4 weeks of treatment with tezacaftor (TEZ)/ivacaftor (IVA), elexacaftor (ELX)/TEZ/IVA, and lumacaftor (LUM)/IVA for patients with complex alleles A238V, I1027T, and L467F, respectively. The 50-year-old patient bearing F508del;A238V/D1152H showed a normal sweat test (13 mEq/L) and improvements in forced expiratory volume in the first second (FEV_1_) (+7 points), body mass index (BMI) (+0.85), and respiratory CF Questionnaire-Revised (CFQ-R) domain (+22.2 points). The 12-year-old patient bearing F508del;I1027T/R709X showed an improvement in a sweat test (−40 mEq/l), FEV_1_ (+9 points) and the respiratory CFQ-R domain (+16.7 points). No changes in outcomes were observed for the 6-year-old patient F508del;L467F/F508del. Our data highlight that the reported variants do not modify the phenotypic expression of F508del. Searching L467F is crucial in CF patients with F508del nonresponsive to ELX/TEZ/IVA. Further data are needed to evaluate the clinical effect of these variants after a longer follow up.

## 1. Introduction

Cystic fibrosis (CF) is the most common, life-limiting, autosomal recessive inherited disease among Caucasians, affecting around 50,000 patients in Europe and 30,000 in the United States. It is caused by variants in the CF transmembrane conductance regulator (*CFTR*) gene. The CFTR protein functions as an ion channel that mediates chloride and bicarbonate transport in the epithelial cells of multiple organs including the lungs, pancreas, and intestine. A dysfunction of the *CFTR* protein induces aberrant ion and fluid homeostasis at epithelial surfaces [1,2]. The phenotype of CF is characterized by elevated sweat chloride levels (SCLs), exocrine pancreatic insufficiency (PI), progressive lung disease with chronic bacterial infections of lower airways, impaired growth, hepatobiliary manifestations, and male infertility [1]. To date, 382 *CFTR* variants are known to cause CF among the 466 reported in the *CFTR2* database (https://cftr2.org, accessed on 20 July 2022). These variants can be categorized into CF-causing variants, variants with varying clinical consequences, non-CF-causing variants, and variants of uncertain significance. Although the frequency of *CFTR* variants differs among populations, the deletion of phenylalanine at position 508 (Phe508del, also known as F508del), is the most prevalent variant, accounting for approximately two-thirds of mutated alleles in the northern European and North American cohorts [3,4]. However, in Italy, less than 50% of CF patients carry the F508del [5] variant. The F508del variant induces misfolding of the protein that is retained in the endoplasmic reticulum and degraded by proteasomal pathways. It also presents defective gating and a considerable reduction in protein stability [6,7].

Substantial efforts have been made in drug development for CF in order to rectify the functional defects caused by these *CFTR* mutations. The drugs that are currently used in treating CF can be divided into two classes of small molecules: potentiators and correctors. The first class of drugs to be successfully developed was *CFTR* potentiators, such as ivacaftor (VX-770, IVA), which are small molecules that interact with the mutant channel to augment its opening probability, enhancing anion flux across the plasma membrane [8,9,10]. *CFTR* correctors, such as lumacaftor (VX-809, LUM), are pharmacological compounds that partially rescue the processing and trafficking defects of the misfolded *CFTR* protein to enable the protein to reach the cell surface. To date, three combinations of *CFTR* correctors and potentiators have reached the market for the treatment of CF patients carrying specific *CFTR* variants. These therapeutic combinations are prescribed based on the type of *CFTR* variant; furthermore, the age of eligible recipients is highly variable in different countries, according to the legislative directives. LUM was shown to rescue F508del-*CFTR* function to approximately 15% of normal channel activity in human bronchial epithelial cells treated in combination with the potentiator IVA (VX-770) [11]. The combination LUM/IVA (Orkambi^®^) is approved in the USA and EU for patients who are homozygous for the F508del variant and who are 2 years of age and older [12]. More recently, the related tezacaftor (VX-661, TEZ)-IVA combination was approved as Symdeko^®^. In the USA, Symdeko^®^ is a prescription treatment for patients who have two copies of the F508del variant, or who have at least 1 of the 154 variants responsive to treatment with TEZ/IVA, and are aged 6 years and older. In the EU, TEZ/IVA (Symkevi^®^) is used in patients with CF homozygous for the F508del variant or heterozygous for the F508del and “residual function” *CFTR*-variant and aged 6 years and older [13,14]. More recently, the combination of *CFTR* correctors, elexacaftor (VX-445, ELX) and TEZ, in combination with IVA, was approved by the FDA as Trikafta^®^ (ELX/TEZ/IVA). In the USA, ELX/TEZ/IVA is a prescription medicine used for patients who have at least one copy of the F508del variant in the *CFTR* gene or one copy of any other of the 177 variants that have shown an in vitro response to the treatment with this triple combination and are aged 6 years and older. In the EU, the combination ELX/TEZ/IVA (Kaftrio^®^) is used in patients with at least one F508del variant in the *CFTR* gene and are aged 12 years and older. The approval for ELX/TEZ/IVA was obtained due to the fast and stable improvements in lung function, the rate of pulmonary exacerbations (PEx), SCL, CF Questionnaire-Revised (CFQ-R) respiratory domain scores, and body mass index (BMI) in comparison with the other drugs that are currently available on the market [15,16,17]. The strong clinical effect of ELX/TEZ/IVA is attributed to the synergistic effect of the two correctors (ELX and TEZ) in rescuing the misfolded *CFTR* protein to the cell membrane. Moreover, many in vitro studies have suggested that ELX/TEZ/IVA is also effective on other class II variants [18,19,20,21].

The detection of variants via *CFTR* gene sequencing creates a challenge due to the lack of clear and univocal genotype–phenotype correlation. Furthermore, the existence of complex alleles remains a challenge in genetic counselling. These complex alleles result from the combination of two or more *CFTR* variants in *cis* (i.e., on the same allele) that usually act as a pathogenic variant, whereas each single variant has only minor or no effect [22,23]. Furthermore, the presence of complex alleles can alter residual *CFTR* protein activity, leading to resistance to targeted therapies [24,25]. These variants are not routinely screened during diagnostic purposes, and their frequency remains uncertain [26].

In this paper, we report the clinical data for three CF patients bearing at least one F508del allele in combination with *cis* variants A238V, I1027T, or L467F alleles before and after treatment with *CFTR* modulators, monitored for four weeks.

## 2. Materials and Methods

All participants provided written informed consent for the anonymous use of data.

The current study is a prospective, observational study, involving patients with CF, carrying a complex allele including the F508del variant. All patients were followed up at the CF center of Florence, Italy. We evaluated the clinical response at baseline and 4 weeks after administration of the *CFTR* modulator drug according to Italian legislative directives.

The main outcome measures included forced expiratory volume in the 1st second (FEV_1_), calculated according to the Global Lung Function Initiative [27], BMI (kg/m^2^), SCL (mmol/L), six-minute walk test (6MWT, m) distance, rate of PEx [7], CFQ-R [28], and treatment-related adverse events. Lung clearance index 2.5 (LCI_2.5_) was determined in the two pediatric patients, in stable conditions, corrected as recently indicated [29], while a chest computed tomography (CT) scan was performed before and after administering the *CFTR* modulator in the adult patient.

The SCLs were tested according to Gibson Cooke method [30] and performed only in the laboratory of the CF Centre of Florence, by a single expert operator, in order to rule out any lack of harmonization [31].

PI was defined based on at least two values of fecal pancreatic elastase lower than 200 μg/g measured outside acute gastrointestinal diseases [32,33].

All clinical data were collected.

## 3. Results

### 3.1. Clinical Characteristics of the Enrolled Patients

Patient 1 was a 50-year-old Caucasian male with congenital bilateral absence of the vas deferens (CBAVD) and acute recurrent pancreatitis. He performed a sweat chloride test at the age of 41 years. His annual evaluation of SCLs persistently resulted in the intermediate range (last value of chloride: 51 mEq/L). *CFTR* gene scanning on this patient (detection rate 98%) revealed three variants: D1152H, F508del, and A238V. Subsequent parental analysis confirmed the presence of D1152H on one allele (inherited from the mother) and the complex allele F508del and A238V on the other allele (inherited from the father). At the age of 46 years, the patient underwent pancreatic enzyme replacement therapy (PERT) and was started for repeated pathological fecal elastase (<50 µg/gr). Widespread bronchiectasis was identified at the last chest computed tomography (CT) scan, performed at 50-year-old, while noncirrhotic liver disease was identified by the liver ultrasound. *Pseudomonas aeruginosa* was detected twice in response to eradicating antibiotic therapy. Diagnosis of CF suggested a multiorgan involvement according to the guidelines for nonscreened populations [34]. The patient had a normal BMI (22.4 kg/m^2^), good respiratory function for age (FEV_1_ before starting therapy: 80%), and required one annual PEx, needing oral antibiotics before starting *CFTR* modulator therapy.

Patient 2 was a 12-year-old Caucasian male diagnosed of having CF with PI for positive newborn screening, according to the protocol used in Tuscany region, Italy [35]. The SCL at diagnosis was pathological (i.e., chloride 103–104 mmol/L). *CFTR* genetic analysis identified three variants: R709X, F508del, and I1027T. The subsequent analysis in the parents confirmed the presence of R709X on one allele (inherited from the mother) and the complex allele F508del/I1027T on the other allele (inherited from the father). The lung disease was classified as moderate (FEV_1_ before starting therapy of 61%) and required one annual PEx, needing oral antibiotics. No bronchiectasis was detected on the chest CT scan, and a normal LCI_2.5_ (6.54 vs. a predicted value of 6.55) was recorded. Annual colonization by methicillin-resistant *Staphylococcus aureus* was detected for this patient from the age of 8 years. Furthermore, he suffered from nasal polyposis, requiring surgery at the age of 5 years and steatosis liver disease at the age of 10 years. The patient’s BMI, before starting modulator therapy, was normal for age (BMI 18.65 kg/m^2^).

Patient 3 was a 6 years and 9 months old Caucasian male, diagnosed of having CF with PI at the age of 19 months, with the presence of respiratory and gastrointestinal symptoms. Newborn screening was not performed in the country of birth. The SCL at diagnosis was pathological (i.e., chloride 105–106 mmol/L). The *CFTR* genetic profile confirmed the presence of variants F508del;L467F/F508del. The main clinical finding for this patient was impaired growth weight (BMI pre-*CFTR* modulator: 14.11 kg/m^2^; 10th percentile), despite PERT. The lung disease was classified as moderate (FEV_1_ 94% at 6 years) by chest bronchiectasis at the upper lung lobes bilaterally, with normal LCI_2.5_ (6.37 vs. a predicted value of 6.55) and two PEx in 2021, requiring oral antibiotics. Furthermore, he suffered from nasal polyposis, requiring surgery at the age of 3 years. No pathogenic CF bacteria were found during the follow-ups.

According to the Italian legislative directives, in January 2022 TEZ-IVA, ELX/TEZ/IVA, and LUM/IVA therapies were prescribed to patients 1, 2, and 3, respectively, without modifying the physiotherapy program or the remaining therapies to which the patients were already subjected. The drugs were taken with fat-containing food to increase their bioavailability [36].

### 3.2. Outcome Measures following CFTR Modulators Treatment

The outcome measures following *CFTR* modulator therapy are reported in Table 1.

Patient 1: After 4 weeks of TEZ-IVA therapy, the sweat test reached a normal value and we observed an improvement in FEV_1_ (+7 points), BMI (+0.85), 6MWT (+27 m), and CFQ-R domains (+ 22.2 points for respiratory domain).

Patient 2: After 4 weeks of ELX/TEZ/IVA therapy, the sweat test and FEV_1_ significantly improved (−40 mEq/L and +9 points, respectively); the BMI value did not change, while nonrelevant drops in LCI (<17%) [37] and 6MWT (+19 m) were observed. Similarly, an improvement in all CFQ-R domains was evident (+16.7 points for respiratory domain) (Table 1).

Patient 3: After 4 weeks of LUM/IVA, no positive change was observed for this patient. Modulator therapy did not change the sweat test result, while FEV_1_ and BMI decreased (−10 points and −0.36, respectively). The LCI value was already normal at baseline (compared with the predicted value of 6.55), due to which less information was detectable after therapy. The CFQ-R domains confirmed a lack of response, due to which the parents requested to evaluate the withdrawal of the drug. However, the child is still continuing the therapy.

No patient required antibiotic therapy during the post-therapy follow-up.

No adverse events leading to interruption of therapy were reported, and no patients withdrew from the treatment. No relevant abnormal results were identified in the clinical laboratory tests, vital signs or physical examinations.

## 4. Discussion

In this study, we identified the CF phenotype in three patients with CF, carrying a complex allele including F508del, and evaluated the clinical effectiveness of treatment with *CFTR* modulators.

Several outcomes can be used to evaluate the effectiveness of *CFTR* modulator drugs [38]. The sweat test is the main biomarker of efficacy in terms of improvement in *CFTR* protein expression and function induced by drugs [39,40,41,42]. Other parameters correlated with lung function are the improvement in the absolute FEV_1_; the 6MWT distance, a simple and well-tolerated test that reflects activities of daily living [43]; and a reduction in the LCI_2.5_ value. This LCI_2.5_ is measured by the multiple-breath washout test. It is a lung function outcome that is more sensitive than spirometry, especially in children, which correlates with the airway changes seen on high-resolution computed tomography and detects significant treatment effects in randomized controlled trials or in children with rare variants [44,45,46,47]. A percentage change in LCI greater than ±15% in preschool children can be considered physiologically relevant and greater than the biological variability in the test [48]; in the same way, a change >17% compared with a previous LCI measurement is a relevant change [37].

Recent studies comparing CT scans findings before initiation of LUM-IVA to one year into LUM-IVA have shown an improvement in mucus plugging, but not in bronchiectasis extent or severity [38,48,49]. Finally, the CFQ-R scores were used to evaluate changes in quality of life, especially focusing on the respiratory domain. In our study, two patients were responders to the modulator drug: the adult patient, carrying the genetic profile F508del;A238V/D1152H, showed early improvements in all clinical parameters and CFQ-R domains. Similar improvement, except regarding nutritional status, was observed for the child carrying the genetic profile F508del;I1027T/R709X and in therapy with ELX/TEZ/IVA. The improvement in the walked distances of these two subjects was not clinically relevant, not being higher than the mean of 54 m previously reported to be associated with a noticeable clinical difference in patients with stable chronic obstructive pulmonary disease [50]. On contrary, the child with the F508del;L467F/F508del genetic profile was a nonresponder, as there was no improvement (or it was not relevant to 6MWT) on the parameters considered.

Genetic variants in *cis* with F508del can affect the phenotype and cause resistance to treatment with *CFTR* modulators. Several complex alleles with F508del have been reported to date, with A238V, L467F, and I148T being the most frequent [22,25,26].

The A238V *CFTR* variant was first described by Picci et al. (http://www.genet.sickkids.on.ca/, accessed on 20 July 2022) in a CBAVD patient from southern Italy, carrying F508del on the other allele. This patient was PI, with an SCL of 60 mmol/L and moderate/mild lung disease. In 2016, a cohort of Italian patients with the A238V;F508del complex allele was described by Diana et al. [51]. In their study, they reanalyzed the *CFTR* genotype of 218 patients, homozygous for F508del (n = 63) or compound heterozygous for the F508del and another variant in the *CFTR* gene (n = 155). They identified the complex allele in 7 (11%) of the patients who were homozygous for F508del and 11 (7%) of the patients who were compound heterozygous for F508del and another variant in the *CFTR* gene. Compared with CF patients homozygous for F508del or compound heterozygous for F508del and another variant, patients with the A238V;F508del complex allele showed a higher amount of C-reactive protein, possibly reflecting a worsened lung inflammation; nevertheless, no corresponding worsening of spirometric parameters such as FEV_1_ was found. Furthermore, the SCLs were lower in the A238V;F508del/other *CFTR* variant than in the F508del/other variant (*p* = 0.03) and less frequent CF-related complications were identified. Interestingly, in vitro studies using heterologous expression systems (i.e., human embryonic kidney (HEK-293) cells) demonstrated no protein processing defect of this *cis* variant (A238V) in comparison with the wild-type (WT) *CFTR* protein. The authors further demonstrated that the A238V variant showed a reduction in A238V-*CFTR* function using yellow fluorescent protein (YFP)-based functional assays. However, the reduced function was not statistically significant, and the reduction was increased by the potentiator IVA [25]. Interestingly, when HEK-293 cells were transfected with double mutants (A238V;F508del), the *CFTR* presented protein processing and functional defect comparable to those of F508del-*CFTR,* which was rescued by the corrector VX-809. In our pilot study, we investigated the effect of TEZ-IVA therapy in an adult patient (number 1 in Table 1) with A238V in F508del complex allele. In agreement with the findings of in vitro studies [25], after a short-term treatment (4 weeks), he showed a sweat chloride level in a normal range (13 mEq/L), a modest lung function (FEV_1_ + 7 points) and improvements in nutritional status and CFQ-R domains.

Another *cis* variant that we investigated was I1027T, which was exclusively in *cis* with the F508del variant and reported in more than 5% of the people in southern Brittany, France [52]. As reported in the CFTR2 database (https://cftr2.org/, accessed on 20 July 2022), I1027T is a variant that does not cause CF clinical characteristics based on the two reported patients worldwide. Moreover, in vitro studies in Fischer rat thyroid (FRT) and CFBE cell lines showed that I1027T-*CFTR* maturation and function are comparable to WT-*CFTR* levels (https://cftr2.org/, accessed on 20 July 2022). In addition, it was demonstrated that while the complex allele F87L;I1027T does not alter WT-*CFTR* maturation, the F508del genetic background (F87L;I1027T;F508del) abolished the VX-809-mediated *CFTR* rescue in HEK-293 cells [25]. Therefore, it would be helpful to understand this *cis* variant’s effect on the F508del variant and predict the *CFTR* modulator’s efficacy in patient-derived tissues (i.e., primary bronchial cells, primary nasal cells, or rectal biopsy-derived organoids).

We also studied the L467F variant in the current study. The L467F;F508del complex allele was reported to be frequent among the Russian population, with a proportion of 4.42% in CF patients [26], similar to the 6% reported by Ivanov et al. [53]. The authors studied the phenotype of 16 CF patients: 5 out of 122 identified between heterozygous for F508del and another *CFTR* variant, while 11 out of 120 were homozygous for the F508del variant. The clinical features of the latter 11 were compared with those of 73 homozygous F508del in absence of a complex allele. No significant differences in clinical parameters or CF related diseases were found between the two groups, even if the mortality was higher in the F508del/F508del group.

The functional effect of L467F on F508del was first studied by Baatlallah et al. in 2017 [25]. When expressed as a single mutant in HEK-293 cells, the L467F variant produced significant reductions in L467F-*CFTR* maturation and function, which were rescued by the corrector VX-809. However, the double mutant F508del;L467F showed a significant reduction in *CFTR* maturation compared with the single mutant of F508del-*CFTR*. Interestingly, the L467F in complex allele with F508del showed no rescue by the LUM/IVA CFTR modulator combination [25]. Similar data were recently confirmed by Sondo et al. for two CF patients followed at the CF center of Genoa, Italy, not responding to ELX/TEZ/IVA therapy, using two different cell models (FRT and CFBE41o-). The results of in vitro studies, using primary nasal epithelial cells from the same CF donors, showed the lack of rescue by ELX/TEZ/IVA similar to the observations during the clinical study. To further investigate the molecular consequences, the authors found that ELX/TEZ/IVA rescued the L467F-CFTR function from 60% to 80% of the activity of WT-CFTR in CFBE cells. ELX/TEZ significantly increased the expression of the mature form of L467F-*CFTR*. L467F in the complex allele with F508del failed to be rescued by ELX/TEX [54]. Despite this being only one subject without corresponding in vitro data, in the current work, our data are in agreement with those of Sondo et al., showing no clinical benefit of ELX/TEZ/IVA in a patient bearing L467F in the complex allele with F508del. Future in-vitro studies are needed to investigate the molecular consequence of *cis* variants in F508del rescued by CFTR modulators. Therefore, taking these studies together, these limiting data suggest the importance of screening for *cis* variants, in particular L467F in F508del, before clinically choosing the drug treatment combination. Moreover, the variability that is observed during ELX/TEZ/IVA treatment in patients that do not respond to the modulator therapy may be due to the *cis* variant, which is not rescuable.

The phenotype of CF patients with *cis* variants in F508del complex allele needs further exploration. Based on our three cases, we hypothesize that these variants (A238V; I148T; L467F) may not alter the protein expression of F508del-CFTR. The clinical presentation of the first adult patient, having D1152H as a second variant, was similar to that of subjects with this variant and a second severe variant in *trans* [55,56,57]. Because the two other patients were children, it was difficult to predict the severity of the disease. However, both children were pancreatic-insufficient during diagnosis, and it is known that the pancreatic status is closely related to the *CFTR* genotype in the case of pediatric patients [58]. Furthermore, we previously reported another child with the F508del;L467F/5T;TG12 *CFTR* genotype [59]. He had asymptomatic CF, transmembrane conductance regulator-related metabolic syndrome CF screen-positive inconclusive diagnosis (CRMS/CFSPID) that progressed to a CF diagnosis at 4 years with a positive sweat test (chloride 65–75 mmol/L), similar to Italian CRMS/CFSPID with F508del/5T;TG12 [5,60]. The clinical behavior of CF patients bearing these variants requires a longer follow-up period in order to obtain a better idea about the effect of these variants in patients.

## 5. Conclusions

The current study highlights the importance of studying the *cis* variants among CF patients using three patients bearing the F508del variant with A238V, I1027T, or L467F alleles. In combination with previous studies, our study also demonstrates the influence of these variants in the differential responses observed in patients toward the *CFTR* modulator therapy combinations. Thus, studying *cis* variants in CF would help in determining potential clinical improvements on a personalized level for CF patients. Upon identifying the different *cis* variants in the *CFTR* gene, it is recommended that patient-specific tissues are studied under in vitro conditions before determining a suitable modulator therapy combination for a CF patient.

## Figures and Tables

**Table 1:** Comparison of change in all variables over the treatment period.

Characteristics	Baseline	After 4 Weeks	Baseline	After 4 Weeks	Baseline	After 4 Weeks
	***Subject 1 °***		***Subject 2 °***		***Subject 3 °***	
Sweat chloride (mEq/L)	51	13	104	64	106	101
FEV_1_ (%)	80	87	61	70	91	81
BMI (kg/m^2^)	22.40	23.25	18.65 ^×^	18.66	14.11 ^~^	13.75
6MWT (m)	630	657	551	570	550	580
LCI_2.5_^∧^	n.a	n.a	7.23	6.69	6.54	6.43
CFQ-R 14+ domain *						
Physical functioning	58.3	91.7	50.0	77.8	72.2	55.6
Role perception	50.0	75.0	/	/	/	/
Vitality	53.3	73.3	/	/	/	/
Emotion	88.9	100.0	83.3	87.5	79.2	79.2
Social perception	22.2	33.3	76.2	81.0	47.6	47.6
Body image	33.3	55.6	100.0	100.0	100.0	100.0
Eating disturbance	44.4	44.4	77.8	100.0	0.0	0.0
Treatment burden	55.6	66.7	22.2	44.4	55.6	55.6
Health perception	41.7	58.3	/	/	/	/
Weight	100.0	100.0	/	/	/	/
Respiratory symptoms	72.2	94.4	83.3	100.0	50.0	50.0
Digestive symptoms	55.6	77.8	66.7	100.0	66.7	66.7

° Patients took TEZ-IVA (subject 1), ELX/TEZ/IVA (subject 2), or LUM/IVA (subject 3) therapies; ^∧^ the predicted value for patients 2 and 3 was 6.55; ^×^ 65th percentile for age; ^~^ 11the percentile for age; * based on patient age (adult, 12–13 years, and 6–11 years, respectively). Abbreviations: FEV1: forced expiratory volume in the 1st second; BMI: body mass index; 6MWT: six-minute walk test; LCI: lung clearance index; CFQ-R: CF Questionnaire-Revised; n.a: not available.

## Data Availability

The data presented in this study are available on request from the corresponding author.
